# Metabolomic profiling and dual antimicrobial and antioxidant activities of <em>Coleus scutellarioides</em> (L.) Benth. leaves against <em>Neisseria gonorrhoeae</em> and <em>Candida albicans</em>: An <em>in vitro</em> study

**DOI:** 10.14202/vetworld.2026.2931-2950

**Published:** 2026-07-11

**Authors:** Marni Br Karo, Farida Mentalina Simanjuntak, Tetty Rina Aritonang, Desweri Muhareni, Dea Dea, Yola Maharani

**Affiliations:** 1 Sekolah Tinggi Ilmu Kesehatan Medistra Indonesia, Bekasi 17113, Indonesia.; 2 Undergraduate Study Program of Pharmacy, Sekolah Tinggi Ilmu Kesehatan Medistra Indonesia, Bekasi 17113, Indonesia.; 3 Undergraduate Study Program of Midwifery, Sekolah Tinggi Ilmu Kesehatan Medistra Indonesia, Bekasi 17113, Indonesia.

**Keywords:** antioxidant activity, Candida albicans, Coleus scutellarioides, genital infections, LC–HRMS, metabolomics, Neisseria gonorrhoeae, phytochemicals

## Abstract

**Background and Aim:**

The increasing antimicrobial resistance of *Neisseria gonorrhoeae* and *Candida albicans* has created an urgent need for alternative therapeutic agents with multiple biological activities. Although *Coleus scutellarioides* (L.) Benth. (Miana) has long been used in traditional medicine to treat infectious diseases; however, comprehensive metabolomic characterization, together with standardized evaluation of its antimicrobial and antioxidant activities against these clinically important pathogens, remains limited. This study aimed to characterize the secondary metabolite profile of ethanol extracts of Miana leaves using untargeted liquid chromatography–high-resolution mass spectrometry (LC–HRMS) and to evaluate their antimicrobial and antioxidant activities.

**Materials and Methods:**

Leaves were extracted by maceration using 96% ethanol. Untargeted metabolomic profiling was performed using LC–HRMS with an Orbitrap Q Exactive Plus system. Antimicrobial activity against *N. gonorrhoeae* and *C. albicans* was evaluated using Clinical and Laboratory Standards Institute-guided broth microdilution assays to determine the minimum inhibitory concentration (MIC) and minimum bactericidal concentration/minimum fungicidal concentration (MBC/MFC). Antioxidant activity was determined using 2,2-diphenyl-1-picrylhydrazyl (DPPH) and 2,2′-azino-bis(3-ethylbenzothiazoline-6-sulfonic acid) (ABTS) radical scavenging assays by calculating the half-maximal inhibitory concentration (IC₅₀).

**Results:**

LC–HRMS detected 243 metabolites, including 20 major bioactive compounds belonging predominantly to flavonoids, diterpenoids, terpenoids, phenolic acids, coumarins, fatty acids, and alkaloids. Major constituents included apigenin, kaempferol, caffeic acid, lupeol, triptolide, carnosol, kahweol, nootkatone, and betaine. The extract demonstrated fungicidal activity against *C. albicans,* with MIC and MFC values of 62,500 ppm, and bactericidal activity against *N. gonorrhoeae,* with MIC and MBC values of 100,000 ppm (MFC/MIC and MBC/MIC ratios = 1.0). Antioxidant evaluation revealed a very strong DPPH scavenging activity (IC₅₀ = 43.68 ppm) and moderate ABTS scavenging activity (IC₅₀ = 128.7 ppm), both exhibiting concentration-dependent inhibition.

**Conclusion:**

Ethanol extracts of Miana leaves contain diverse bioactive metabolites that confer dual antimicrobial activity against *N. gonorrhoeae* and *C. albicans* tog ether with potent antioxidant activity. These findings provide a comprehensive phytochemical foundation that supports the therapeutic potential of Miana and justifies further bioassay-guided fractionation, toxicity assessment, mechanistic investigations, and *in vivo* validation to facilitate the development of standardized phytopharmaceutical agents for the management of genital infections.

## INTRODUCTION

Antimicrobial resistance is one of the greatest global public health challenges, accounting for an estimated 4.95 million deaths annually, with projections indicating that mortality could reach 10 million deaths per year by 2050 if effective interventions are not implemented [1–5]. *Neisseria gonorrhoeae* and *Candida albicans* are priority pathogens identified by the World Health Organization because of their increasing resistance to conventional antibiotics and antifungal agents, which has intensified the global burden of sexually transmitted infections and vulvovaginal candidiasis, affecting approximately 87 million and 138 million individuals annually, respectively [6–8]. *N. gonorrhoeae* has developed resistance to penicillin, tetracycline, fluoroquinolones, and third-generation cephalosporins, whereas *C. albicans* has acquired resistance to azoles and echinocandins through multiple mechanisms, including target-site mutations, efflux pump overexpression, and biofilm formation [9–12]. Coinfections involving these pathogens frequently occur in the reproductive tract, manifesting clinically as urethritis, cervicitis, and vulvovaginitis, and often require combined antibacterial and antifungal therapy. However, such treatment may increase the risk of adverse drug reactions, drug interactions, treatment costs, and the continued emergence of antimicrobial resistance [6]. Consequently, there is increasing interest in identifying plant-derived therapeutic agents possessing multiple mechanisms of action that may serve as effective alternatives or complementary therapies to conventional antimicrobial agents [13, 14].

Oxidative stress also plays an important role in the pathogenesis of genital infections. Excessive production of reactive oxygen species (ROS) promotes tissue damage, disrupts epithelial integrity, and weakens local immune defenses, thereby facilitating pathogen persistence and disease progression. Plant-derived antioxidant compounds may provide dual therapeutic benefits by directly inhibiting microbial growth while simultaneously reducing oxidative tissue injury [15–21]. Therefore, evaluating both the antimicrobial and antioxidant activities of medicinal plants is essential for developing multifunctional therapeutic agents that eliminate pathogens while preserving host tissue integrity. Indonesia possesses exceptional plant biodiversity, with more than 30,000 plant species, approximately 7,000 of which are recognized for their medicinal properties and have traditionally been used to treat various infectious diseases [22, 23]. Among these medicinal plants, *Coleus scutellarioides* (L.) Benth. (synonyms: *Coleus blumei*, *Plectranthus scutellarioides*, and *Solenostemon scutellarioides*), commonly known as Miana or jawer kotok, belongs to the family Lamiaceae and has long been used in traditional medicine to treat cough, fever, diarrhea, skin infections, and respiratory disorders [24–27]. Phytochemical investigations have demonstrated that Miana leaves contain flavonoids, terpenoids, alkaloids, saponins, tannins, and essential oils exhibiting antibacterial, antifungal, anti-inflammatory, and antioxidant activities [15–21]. Tarigan *et al.* [17] reported that methanolic extracts of Miana leaves inhibited the growth of *Staphylococcus aureus*, whereas Bismelah *et al.* [28] demonstrated antibacterial activity against periodontal bacteria, reporting a minimum inhibitory concentration (MIC) of 1.56 mg/mL and a minimum fungicidal concentration (MFC) of 3.13 mg/mL.

Despite these promising findings, several important knowledge gaps remain. Previous studies have largely relied on conventional phytochemical techniques, such as thin-layer chromatography and spectrophotometry, which provide only limited information regarding the comprehensive metabolite composition of Miana. Furthermore, most investigations have focused primarily on gram-positive bacteria, oral pathogens, or dermatophytes and have not evaluated clinically important sexually transmitted pathogens, such as *N. gonorrhoeae* and *C. albicans,* using standardized Clinical and Laboratory Standards Institute (CLSI)-based antimicrobial susceptibility testing methods. Although the antioxidant properties of Miana leaves have been independently reported by Ślusarczyk *et al.* [15] and Pakadang *et al.* [20], these activities have not been systematically investigated alongside antimicrobial activity within a single, integrated experimental framework. Moreover, although previous studies have demonstrated *in vivo* antifungal effects against *C. albicans* in murine vulvovaginal infection models and antibacterial activity against oral and gram-positive pathogens, no published study has comprehensively combined untargeted metabolomic profiling with standardized *in vitro* MIC and MFC determination against both *N. gonorrhoeae* and *C. albicans*. Consequently, the relationships between the metabolomic composition of Miana extracts and their dual antimicrobial and antioxidant activities remain poorly understood, thereby limiting the scientific evidence needed to develop standardized phytopharmaceutical products.

Therefore, this study aimed to comprehensively characterize the secondary metabolite profile of ethanol extracts of Miana leaves using untargeted liquid chromatography–high-resolution mass spectrometry (LC–HRMS). In addition, the antimicrobial activities of the extracts against *N. gonorrhoeae* and *C. albicans* were evaluated by determining the MIC and MFC values using the standardized CLSI broth microdilution method, while antioxidant activity was assessed using 2,2-diphenyl-1-picrylhydrazyl (DPPH) and 2,2′-azino-bis(3-ethylbenzothiazoline-6-sulfonic acid) (ABTS) radical scavenging assays. Untargeted LC–HRMS enables simultaneous identification of diverse secondary metabolites with high mass accuracy (<5 ppm) and compound-level annotation confidence in accordance with the Metabolomics Standards Initiative guidelines, thereby providing a comprehensive phytochemical profile that supports extract standardization and structure–activity relationship analyses [29]. The selection of *N. gonorrhoeae* and *C. albicans* as test microorganisms is clinically relevant because both pathogens are major causes of genital infections, frequently coinfect, and exhibit increasing antimicrobial resistance, often necessitating combination therapy [30, 31]. The findings of this study are expected to provide robust scientific evidence supporting the dual antimicrobial and antioxidant potential of Miana leaf extracts, identify bioactive metabolites associated with these biological activities, and establish a comprehensive phytochemical foundation for the future development of standardized phytopharmaceutical agents for the management of gonorrhea, candidiasis, and other genital infections.

## MATERIALS AND METHODS

### Ethical approval

The study protocol was reviewed and approved by the Health Research Ethics Committee of Bakti Tunas Husada University, Tasikmalaya, Indonesia (approval no. 314-01/E.01/KEPK-BTH/IX/2024; approved on September 25, 2024). As this was a laboratory-based *in vitro* study using authenticated plant material and reference microbial strains, no human participants or animals were involved, and informed consent was not required. All experimental procedures involving *N. gonorrhoeae* ATCC 43069P and *C. albicans* ATCC 10231 were conducted in accordance with institutional biosafety requirements and relevant national and institutional laboratory safety guidelines. The microbial cultures were handled only by trained personnel in controlled laboratory facilities, and all contaminated materials, culture media, and biological waste were sterilized before disposal. The study was conducted in accordance with the approved protocol and did not involve the collection of clinical specimens, patient data, or animal-derived samples.

### Study period and location

The study was conducted between January and March 2024 at the Microbiology Laboratory and Phytochemistry Laboratory, Medistra Indonesia College of Health Sciences, Bekasi, West Java, Indonesia. All experimental procedures were performed under controlled laboratory conditions at room temperature (25 ± 2°C) and a relative humidity of 60 ± 5%.

### Study design

This laboratory-based experimental study employed an *in vitro* approach to evaluate the antibacterial, antifungal, and antioxidant activities of ethanol extracts prepared from Miana leaves. Untargeted metabolomic profiling was performed to characterize the extract's phytochemical composition prior to evaluation of biological activity.

### Sample preparation and extraction

Fresh Miana leaves were sorted, thoroughly washed with running tap water, and dried in the shade at ambient temperature (25 ± 2°C) for 7 days until a constant weight was reached. The dried leaves were ground using an electric blender and passed through a 60-mesh sieve to obtain a uniform fine powder.

A total of 200 g of powdered leaves was macerated with 2,000 mL of 96% ethanol (1:10, w/v) in a sealed amber glass container at 25°C for 48 h with intermittent stirring. The macerate was filtered through Whatman No. 1 filter paper, and the plant residue was remacerated twice with fresh solvent until the filtrate became colorless [32]. All filtrates were combined and concentrated using a rotary vacuum evaporator (IKA Werke GmbH & Co. KG, Staufen, Germany) at 40°C under a pressure of 175 mbar until a thick extract of constant weight was obtained. The extraction yield was calculated as the ratio of the weight of the concentrated extract to the initial dry weight of the plant material. The concentrated extract was transferred into sealed amber glass containers and stored at 4°C until further analysis.

The extraction process yielded 28.4 g of thick extract from 200 g of dried leaf powder, corresponding to an extraction yield of 14.2% (w/w). No phytochemical marker-based standardization was performed for this extract batch; however, batch consistency will be evaluated in future studies using LC–HRMS chemical fingerprinting.

### Materials and equipment

Fresh Miana leaves were collected from a medicinal plant garden in Bekasi, West Java, Indonesia. Botanical authentication was performed at the Bogor Herbarium, National Research and Innovation Agency, Bogor, Indonesia, under voucher specimen no. 017/BH/VI/2024.

The test microorganisms used in this study were *N. gonorrhoeae* (American Type Culture Collection [ATCC] 43069P) and *C. albicans* (ATCC 10231), obtained from the ATCC, Manassas, VA, USA.

The chemicals used in this study included 96% ethanol, LC–MS-grade methanol, chocolate agar medium (HiMedia Laboratories Pvt. Ltd., Mumbai, India), Sabouraud dextrose agar (SDA; HiMedia), Sabouraud dextrose broth (SDB; HiMedia), and 0.9% sodium chloride solution (Otsuka Pharmaceutical Co., Ltd., Tokyo, Japan). McFarland Standard No. 3 (Thermo Fisher Scientific Remel, Lenexa, KS, USA), dimethyl sulfoxide (DMSO), formic acid, and acetonitrile were also used. Vancomycin and nystatin supplied by the Indonesian Food and Drug Authority (BPOM), Jakarta, Indonesia, served as positive controls for antibacterial and antifungal assays, respectively.

The principal instruments included a UV–Vis spectrophotometer (B-ONE, Shanghai, China), a biological safety cabinet (Medfuture, Munich, Germany), a rotary vacuum evaporator (IKA Werke GmbH & Co. KG, Staufen, Germany), an LC–HRMS system comprising a Q Exactive Plus Orbitrap mass spectrometer coupled with a Dionex UltiMate 3000 liquid chromatography system (Thermo Fisher Scientific), and an Acclaim RSLC 120 C18 analytical column (2.2 μm × 2.1 × 100 mm; Thermo Fisher Scientific). Additional laboratory equipment included an incubator (Memmert GmbH + Co. KG, Schwabach, Germany), an autoclave (GEA, Guangzhou, China), sterile 96-well microplates, and borosilicate laboratory glassware (Pyrex, Corning, NY, USA).

### Metabolomic analysis via LC–HRMS

Untargeted LC–HRMS metabolomic analysis was performed to characterize the secondary metabolite profile of the Miana leaf extract and identify its bioactive constituents [32]. Briefly, 10 mg of concentrated extract was dissolved in 1 mL of LC–MS-grade methanol, sonicated for 15 min, and centrifuged at 24,562 × *g* for 10 min at 4°C. The supernatant was filtered through a 0.22-μm polytetrafluoroethylene syringe filter before injection into the LC–HRMS system. Three technical replicates of the injection were prepared for each sample. Solvent blanks consisting of LC–MS-grade methanol were processed under identical chromatographic conditions and subsequently subtracted from the chromatograms to eliminate background signals.

Peak processing was performed using Compound Discoverer version 3.3 (Thermo Fisher Scientific). The processing parameters included a minimum peak intensity threshold of 1 × 10⁵, a mass tolerance of 5 ppm, a signal-to-noise ratio of ≥3, and targeted adducts of [M+H]⁺, [M+Na]⁺, and [M+NH₄]⁺ in positive ionization mode and [M−H]⁻ and [M+HCOO]⁻ in negative ionization mode.

Chromatographic separation was achieved using an Acclaim RSLC 120 C18 analytical column (2.2 μm, 2.1 × 100 mm) maintained at 40°C with an injection volume of 5 μL. The mobile phase consisted of (A) water containing 0.1% formic acid and (B) acetonitrile containing 0.1% formic acid. Gradient elution was programmed as follows: 0–2 min, 5% B; 2–20 min, 5%–100% B; 20–25 min, 100% B; 25–26 min, 100%–5% B; and 26–30 min, 5% B at a flow rate of 0.3 mL/min.

Mass spectrometric analysis was performed in both positive and negative ionization modes over an *m/z* range of 100–1500. The electrospray ionization source parameters were set as follows: spray voltage, 3.5 kV; capillary temperature, 320°C; sheath gas, 40 arbitrary units; auxiliary gas, 10 arbitrary units; and mass resolution, 70,000 full width at half maximum.

LC–HRMS data were processed using Compound Discoverer version 3.3 (Thermo Fisher Scientific) for peak detection, spectral deconvolution, and compound annotation using the mzCloud, ChemSpider, and Kyoto Encyclopedia of Genes and Genomes databases. Compound identification was based on mass accuracy (<5 ppm), MS/MS fragmentation patterns, and comparisons with published literature. Compounds with a confidence level of ≥3, as defined by the Metabolomics Standards Initiative, were selected for further interpretation [29]. Both positive and negative electrospray ionization modes were used to maximize metabolite coverage. The positive mode predominantly generated [M+H]⁺ and [M+Na]⁺ adducts, whereas the negative mode primarily detected [M−H]⁻ adducts for phenolic and organic acids. Among the 243 annotated compounds, the 20 major compounds listed in Table 1 were selected based on their signal intensities and established biological relevance, with a Metabolomics Standards Initiative confidence level of ≥3. The complete annotated dataset, including *m/z* values, retention times (RT), adduct types, molecular formulas, and Metabolomics Standards Initiative confidence levels for all detected compounds, is provided in Supplementary Table S1**.**

### Preparation of test microorganisms

*N. gonorrhoeae* cultures were revived on chocolate agar medium and incubated at 37°C in a 5% CO₂ atmosphere for 24 h. *C. albicans* cultures were revived on SDA and incubated aerobically at 37°C for 48 h. Pure colonies were aseptically collected using a sterile inoculating loop and suspended in sterile 0.9% sodium chloride solution to achieve turbidity equivalent to McFarland Standard No. 3 (3 × 10⁸ colony-forming units [CFU]/mL), as determined spectrophotometrically at 625 nm [33].

The microbial suspension (3 × 10⁸ CFU/mL) was serially diluted in sterile 0.9% sodium chloride solution to obtain a working inoculum of 1 × 10⁶ CFU/mL using a 1:100 dilution. Briefly, 10 μL of the 3 × 10⁸ CFU/mL suspension was transferred into 990 μL of sterile 0.9% sodium chloride solution to produce the working suspension. *N. gonorrhoeae* cultures were maintained at 37°C under a 5%–7% CO₂ atmosphere with ≥95% relative humidity. The viability of the working inoculum was confirmed by plate counting on the appropriate solid medium before each experiment, and all inocula were within the acceptable target range.

### MIC assay

The MIC was determined using the broth microdilution method in sterile 96-well microplates in accordance with CLSI guidelines [34]. For antifungal susceptibility testing, the assay followed the CLSI M27 reference standard using SDB [35]. For antibacterial susceptibility testing against *N. gonorrhoeae*, broth microdilution was employed as a high-throughput adaptation, and all inhibitory endpoints were confirmed by subculturing onto chocolate agar because agar dilution remains the CLSI reference method (CLSI M07/M100) for this fastidious organism [36]. This methodological adaptation is recognized as a limitation of the present study.

A final inoculum concentration of 3 × 10⁶ CFU/mL was used to increase the sensitivity of crude extract screening. This concentration exceeded the CLSI-recommended inoculum of 5 × 10⁵ CFU/mL for standard broth microdilution and may therefore have contributed to the relatively high MIC values obtained. This limitation should be considered when interpreting the extract's antimicrobial activity. All microplates were sealed with sterile parafilm before incubation to minimize evaporation.

The Miana leaf extract was dissolved in 10% DMSO and serially diluted with SDB to obtain final concentrations of 250,000, 125,000, 62,500, 31,250, 15,625, and 7,812.5 ppm for antifungal testing. For antibacterial testing, concentrations of 200,000, 100,000, 50,000, 25,000, 12,500, and 6,250 ppm were prepared.

Each well received 100 μL of the extract solution and 100 μL of the microbial suspension (final inoculum, 3 × 10⁶ CFU/mL). Nystatin (0.5–4 ppm) served as the positive control for *C. albicans*, whereas vancomycin (0.5–2 ppm) served as the positive control for *N. gonorrhoeae*. Negative control wells contained sterile medium without microorganisms, whereas growth control wells contained microbial suspension without extract or antimicrobial agents.

The microplates were incubated at 37°C for 48 h under aerobic conditions for *C. albicans* and in a 5% CO₂ atmosphere for *N. gonorrhoeae*. The MIC was defined as the lowest extract concentration showing no visible turbidity compared with the growth control. All experiments were performed in duplicate. When duplicate wells produced concordant results, the lowest concentration showing complete inhibition of visible growth was recorded as the MIC. When discordant results occurred between duplicate wells, the higher (more conservative) concentration was accepted as the MIC in accordance with the CLSI M07-A11 recommendations [37].

To eliminate solvent-related effects, the extract stock solution prepared in 10% DMSO was serially diluted with SDB or chocolate broth so that the final DMSO concentration in each assay well did not exceed 1% (v/v), a concentration reported to have no inhibitory effect on the test microorganisms [38]. At each concentration, the extract solution was visually inspected and examined under light microscopy to verify complete dissolution and the absence of precipitation before inoculation. No visible precipitation was observed at concentrations up to 250,000 ppm (250 mg/mL). In addition, the pH of the medium containing the highest extract concentration differed by no more than ±0.2 units from that of the untreated control, confirming the absence of significant pH-related nonspecific effects. Vehicle control wells containing 1% DMSO alone were included in every assay and demonstrated no inhibition of microbial growth.

### MFC assay

The MFC for *C. albicans* and the minimum bactericidal concentration (MBC) for *N. gonorrhoeae* were determined by subculturing 10-μL aliquots from all wells showing no visible turbidity in the MIC assay onto solid media using the quadrant streak technique. Antifungal subcultures were inoculated onto SDA, whereas antibacterial subcultures were inoculated onto chocolate agar.

The culture plates were incubated at 37°C for 48 h under the same atmospheric conditions as those used in the corresponding MIC assay. The MFC or MBC was defined as the lowest concentration producing no visible colony growth or a maximum of one to two colonies, corresponding to ≥99.9% killing of the initial microbial inoculum [39].

All determinations were performed in duplicate to confirm reproducibility. The lowest concentration that resulted in complete or near-complete elimination of viable microorganisms was recorded as the MFC for *C. albicans* and the MBC for *N. gonorrhoeae*, respectively.

### Quality control

Each antimicrobial susceptibility assay included appropriate quality control measures to ensure the reliability and reproducibility of the results. The following controls were included in parallel with the test samples: (a) a growth control containing the test microorganism in culture medium without extract or antimicrobial agent to confirm microbial viability throughout the assay; (b) a sterility control consisting of culture medium only to verify the absence of contamination; (c) a vehicle control containing the test microorganism in culture medium supplemented with the maximum final concentration of DMSO (≤1%, v/v) to confirm that the solvent did not affect microbial growth; and (d) positive controls comprising nystatin (0.5–4 ppm) for *C. albicans* and vancomycin (0.03–2 ppm) for *N. gonorrhoeae* to verify assay performance during each experimental run.

### Antioxidant activity assay

The antioxidant activity of the Miana leaf extract was evaluated using the ABTS and DPPH radical scavenging assays.

For the ABTS assay, the ABTS•⁺ radical solution was prepared by mixing 7 mM ABTS with 2.45 mM potassium persulfate (1:1, v/v), then incubated in the dark at 25°C for 16 h. The resulting radical solution was diluted with methanol to obtain an absorbance of 0.70 ± 0.02 at 734 nm. The Miana leaf extract was dissolved in methanol to obtain concentrations of 3.91, 7.81, 15.62, 31.25, 62.5, 125, 250, and 500 ppm. Subsequently, 100 μL of each extract solution was mixed with 1,900 μL of the ABTS•⁺ radical solution, vortexed for 30 s, and incubated at 25°C for 6 min in the dark. The absorbance was measured at 734 nm using the UV–Vis spectrophotometer. Trolox served as the positive control, whereas methanol without extract or standard was used as the blank.

For the DPPH assay, a 0.1 mM DPPH radical solution was prepared in methanol. The Miana leaf extract was prepared at concentrations of 3.91, 7.81, 15.62, 31.25, 62.5, 125, 250, 500, 750, 1,000, and 1,500 ppm. A total of 100 μL of each extract solution was mixed with 3,900 μL of the DPPH solution, vortexed for 30 s, and incubated at 25°C in the dark for 30 min. The absorbance was measured at 517 nm using the UV–Vis spectrophotometer. Ascorbic acid was used as the positive control.

The percentage of radical scavenging activity was calculated using the following equation:


\[
Radical inhibition (\%)=\frac{{A}_{control}-{A}_{sample}}{{A}_{control}}\times 100
\]

The half-maximal inhibitory concentration (IC₅₀) values were determined by nonlinear regression using the inhibitor versus normalized response-variable slope model, consistent with the analytical approach used for the ABTS assay.

### Statistical analysis

The MIC, MFC, and MBC values were determined as categorical endpoints based on visual assessment of broth turbidity and confirmation by subculture on solid media. Because these endpoints follow the CLSI broth microdilution methodology, parametric inferential statistical analyses were not applicable, and the antimicrobial susceptibility results are presented descriptively [37]. When duplicate determinations yielded discordant results, the higher (more conservative) concentration was recorded as the MIC.

The antioxidant activities were expressed as IC₅₀ values with corresponding 95% confidence intervals (CI). The relationship between extract concentration and radical-scavenging activity was evaluated using Pearson's correlation coefficient, with statistical significance set at p < 0.05**.**

Microbial growth observations were recorded qualitatively as positive (+) when visible turbidity or colony growth was present and negative (−) when no microbial growth was observed. The inhibitory activities of the Miana leaf extract and the positive controls were compared descriptively according to their effective concentration ranges.

Metabolomic data generated by LC–HRMS were evaluated using chemometric analysis based on principal component analysis. Representative photographs of microbial growth on solid media were captured to document the experimental findings. Dose–response curves for antioxidant activity were generated by nonlinear regression using the inhibitor versus normalized response variable slope model to characterize the relationship between extract concentration and radical-scavenging activity. Statistical significance was accepted at p < 0.05**.**

## RESULTS

### Yield of the ethanol extract of Miana leaves

Maceration of Miana leaves using 96% ethanol produced a thick, dark-green extract with a semisolid consistency. A total of 28.4 g of concentrated extract was obtained from 200 g of dried leaf powder, corresponding to an extraction yield of 14.2% (w/w). The extract exhibited a characteristic aromatic odor and a sticky texture at 25°C.

### Bioactive compounds identified in the ethanol extract of Miana leaves by LC–HRMS

**Figure 1:**
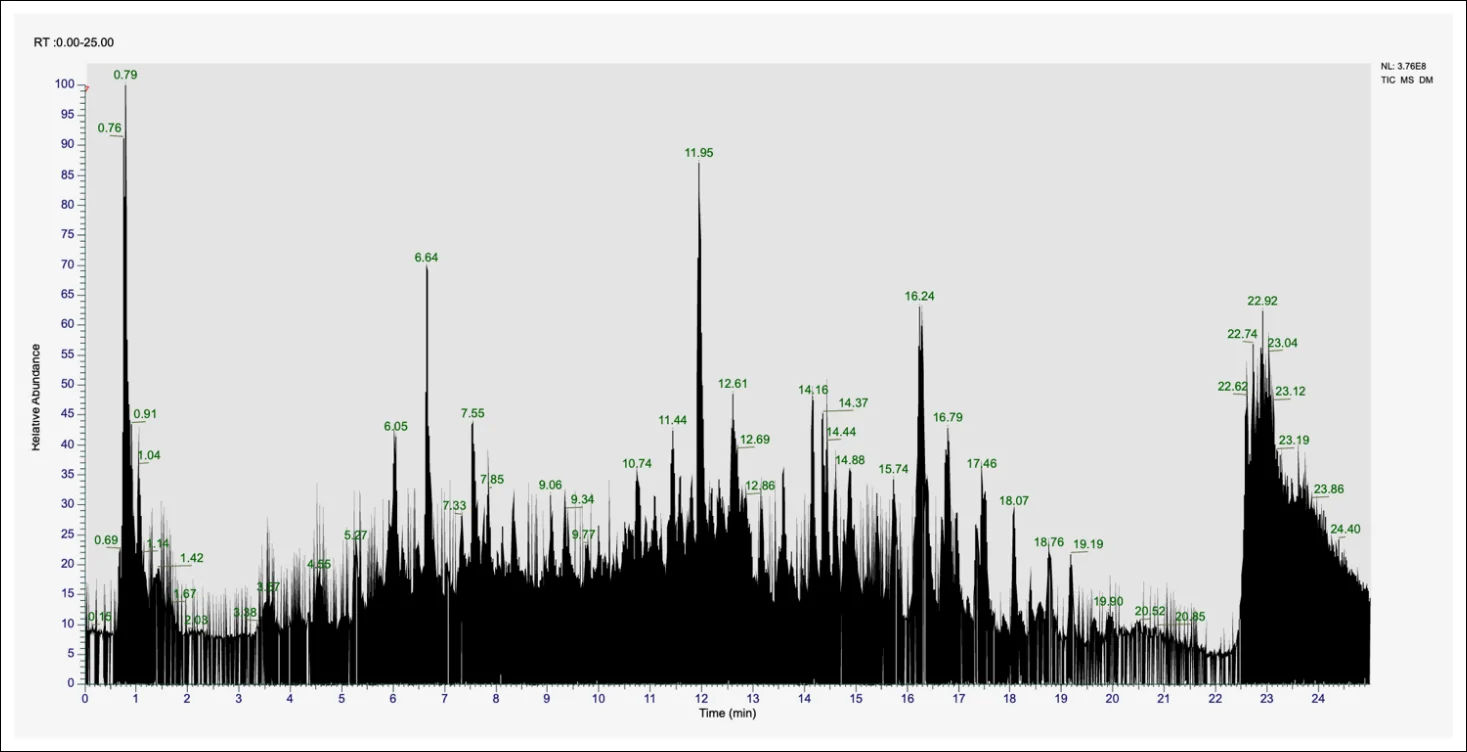
Total ion chromatogram of the ethanol extract of Miana leaves obtained by liquid chromatography–high-resolution mass spectrometry.

Untargeted LC–HRMS metabolomic analysis identified 243 compounds in the ethanol extract of Miana leaves. The total ion chromatogram obtained in positive ionization mode is presented in Figure 1, with major peaks detected at retention time (RT, min) of 0.79, 6.64, 11.95, 16.24, and 22.92 min. Based on mass accuracy, molecular formula assignment, and spectral database matching, 20 major bioactive compounds were annotated with high confidence. These compounds were selected according to their signal intensities and a Metabolomics Standards Initiative confidence level of ≥3. The complete annotated dataset for all 243 compounds detected in both positive and negative ionization modes, including RT, measured *m/z* values, adduct types, molecular formulas, and confidence levels, is provided in Supplementary Table S1.

Table 1 summarizes the 20 major secondary metabolites identified in the ethanol extract of Miana leaves based on signal intensity and biological relevance. Flavonoids constituted the predominant class of metabolites, including apigenin (270.05257 Da; RT, 8.356 min), kaempferol (286.04726 Da; RT, 7.566 min), apigetrin (432.10570 Da; RT, 6.428 min), and cynaroside (448.10024 Da; RT, 5.287 min). Organic acids detected in appreciable amounts included caffeic acid (180.04198 Da; RT, 6.666 min), stearidonic acid (276.20867 Da; RT, 13.641 min), and α-eleostearic acid (278.22417 Da; RT, 14.124 min). The identified terpenoid compounds comprised lupeol (426.38582 Da; RT, 17.695 min), (+)-nootkatone (218.16658 Da; RT, 12.543 min), β-ionone (192.15123 Da; RT, 12.283 min), and (+)-ar-turmerone (216.15111 Da; RT, 12.862 min). Multiple coumarin derivatives (146.03664 Da) were detected at RT of 7.306, 7.086, 6.071, 5.910, 5.738, 5.365, and 4.366 min, indicating the presence of coumarin isomers or structurally related derivatives.

Additional bioactive diterpenoids, including triptolide (360.15679 Da; RT, 11.560 min), carnosol (330.18260 Da; RT, 6.212 min), and kahweol (314.18782 Da; RT, 15.172 min), were also identified. Among the detected metabolites, betaine (RT, 0.79 min), coumarin (RT, 6.64 min), kaempferol (RT, 11.95 min), and lupeol (RT, 22.92 min) exhibited the highest relative signal intensities in the chromatogram. Flavonoid glycosides, including apigetrin and cynaroside, eluted earlier than their corresponding aglycones, apigenin and kaempferol, because of their greater polarity. In contrast, highly hydrophobic terpenoid compounds, such as lupeol, eluted during the final phase of chromatographic separation (RT, 17.695 min), consistent with their physicochemical properties.

**Table 1:** Major secondary metabolites identified in the ethanol extract of Miana leaves by liquid chromatography–high-resolution mass spectrometry.

**No.**	**Compound**	**Molecular formula**	**Structure**	**Calculated MW (Da)**	**RT (min)**	**Compound group**
1	Apigenin	C₁₅H₁₀O₅		270.05257	8.356	Flavonoid
2	Kaempferol	C₁₅H₁₀O₆		286.04726	7.566	Flavonoid
3	Caffeic acid	C₉H₈O₄		180.04198	6.666	Phenolic acid
4	Apigetrin	C₂₁H₂₀O₁₀		432.10570	6.428	Flavonoid glycoside
5	Cynaroside	C₂₁H₂₀O₁₁		448.10024	5.287	Flavonoid glycoside
6	Lupeol	C₃₀H₅₀O		426.38582	17.695	Triterpenoid
7	Stearidonic acid	C₁₈H₂₈O₂		276.20867	13.641	Unsaturated fatty acid
8	α-Eleostearic acid	C₁₈H₃₀O₂		278.22417	14.124	Unsaturated fatty acid
9	Triptolide	C₂₀H₂₄O₆		360.15679	11.560	Diterpenoid
10	(+)-Nootkatone	C₁₅H₂₂O		218.16658	12.543	Sesquiterpenoid
11	β-Ionone	C₁₃H₂₀O		192.15123	12.283	Terpenoid
12	(+)-ar-Turmerone	C₁₅H₂₀O		216.15111	12.862	Sesquiterpenoid
13	Kahweol	C₂₀H₂₆O₃		314.18782	15.172	Diterpenoid
14	Carnosol	C₂₀H₂₆O₄		330.18260	6.212	Diterpenoid
15	Coumarin	C₉H₆O₂		146.03664	7.306	Coumarin
16	Diosmetin	C₁₆H₁₂O₆		300.06328	**8.514**	Flavonoid
17	Tangeretin	C₂₀H₂₀O₇		372.12029	10.721	Methylated flavonoid
18	Erucamide	C₂₂H₄₃NO		337.33370	17.204	Fatty acid amide
19	Methyl palmitate	C₁₇H₃₄O₂		270.25566	17.048	Fatty acid ester
20	Betaine	C₅H₁₁NO₂		117.07902	0.801	Alkaloid

MW = Molecular weight, RT = Retention time (min). Chemical structures were validated using the PubChem database.

### Antimicrobial activity of the ethanol extract of Miana leaves

**Figure 2:**
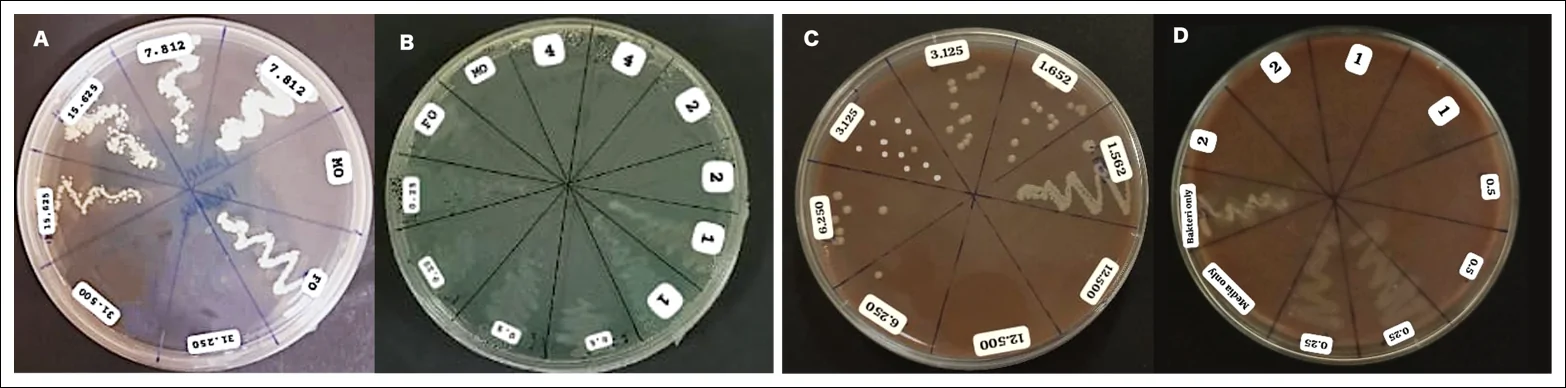
Confirmation of the antimicrobial activity of the ethanol extract of Miana leaves on solid media. (A) Growth of *Candida albicans* on Sabouraud dextrose agar following treatment with various concentrations of the Miana leaf extract and nystatin (positive control). (B) Growth of *Neisseria gonorrhoeae* on chocolate agar following treatment with various concentrations of the Miana leaf extract and vancomycin (positive control). MO = Medium only (negative medium control); BO = Microorganism only (growth control). No colony growth was observed at extract concentrations of ≥62,500 ppm for *C. albicans* and ≥100,000 ppm for *N. gonorrhoeae*.

The ethanol extract of Miana leaves exhibited both antifungal and antibacterial activities against the tested pathogens (Table 2). Against *C. albicans*, the extract produced an MIC of 62,500 ppm (62.5 mg/mL) and an MFC of 62,500 ppm (62.5 mg/mL), yielding an MFC/MIC ratio of 1.0, indicating fungicidal activity. Visual examination of the microplates revealed no turbidity at extract concentrations of 250,000, 125,000, and 62,500 ppm, whereas visible turbidity was observed at concentrations of ≤31,250 ppm. Subculture on SDA confirmed the absence of fungal colony growth at concentrations of ≥62,500 ppm (Figure 2A).

The extract also demonstrated antibacterial activity against *N. gonorrhoeae*, with both the MIC and MBC determined to be 100,000 ppm (100 mg/mL), yielding an MBC/MIC ratio of 1.0 and confirming bactericidal activity. No visible turbidity was observed at extract concentrations of 200,000 and 100,000 ppm. At 50,000 ppm, one replicate showed complete inhibition of bacterial growth, whereas the second replicate exhibited visible turbidity. In accordance with CLSI recommendations, the higher concentration (100,000 ppm) was therefore designated as the MIC. At the reported MIC values (62,500 ppm for *C. albicans* and 100,000 ppm for *N. gonorrhoeae*), duplicate wells produced concordant results with no visible turbidity. Bacterial growth was clearly observed at concentrations of ≤25,000 ppm. Subculture on chocolate agar confirmed the absence of bacterial colony growth at concentrations of ≥100,000 ppm (Figure 2B).

**Table 2:** Antimicrobial activity of the ethanol extract of Miana leaves against the test pathogens.

**Pathogen**	**Sample**	**MIC (ppm/mg/mL)**	**MFC/MBC (ppm/mg/mL)**	**Ratio**	**Activity type**
Candida albicans	Miana extract	62,500/62.5	62,500/62.5	1.0	Fungicidal
	Nystatin*	2/0.002	2/0.002	1.0	Fungicidal
*Neisseria gonorrhoeae*	Miana extract	100,000/100	100,000/100	1.0	Bactericidal
	Vancomycin†	0.0625/0.0000625	0.0625/0.0000625	1.0	Bactericidal

*Positive control for the antifungal assay; †Positive control for the antibacterial assay. MIC = Minimum inhibitory concentration; MFC = Minimum fungicidal concentration; MBC = Minimum bactericidal concentration.

The positive control nystatin inhibited *C. albicans* at an MIC and MFC of 2 ppm, whereas vancomycin inhibited *N. gonorrhoeae* at an MIC and MBC of 0.0625 ppm. Compared with the positive controls, the antimicrobial activity of the Miana leaf extract was approximately 31,250-fold lower than that of nystatin and 1,600,000-fold lower than that of vancomycin. Nevertheless, the identical MFC/MIC and MBC/MIC ratios (1.0) observed for both the extract and the positive controls indicate that they produced fungicidal and bactericidal effects, respectively, at their corresponding MICs.

The negative medium control remained free of contamination throughout the experiment, whereas the microbial growth control demonstrated satisfactory viability during the incubation period.

### Antioxidant activity of the ethanol extract of Miana leaves

**Figure 3:**
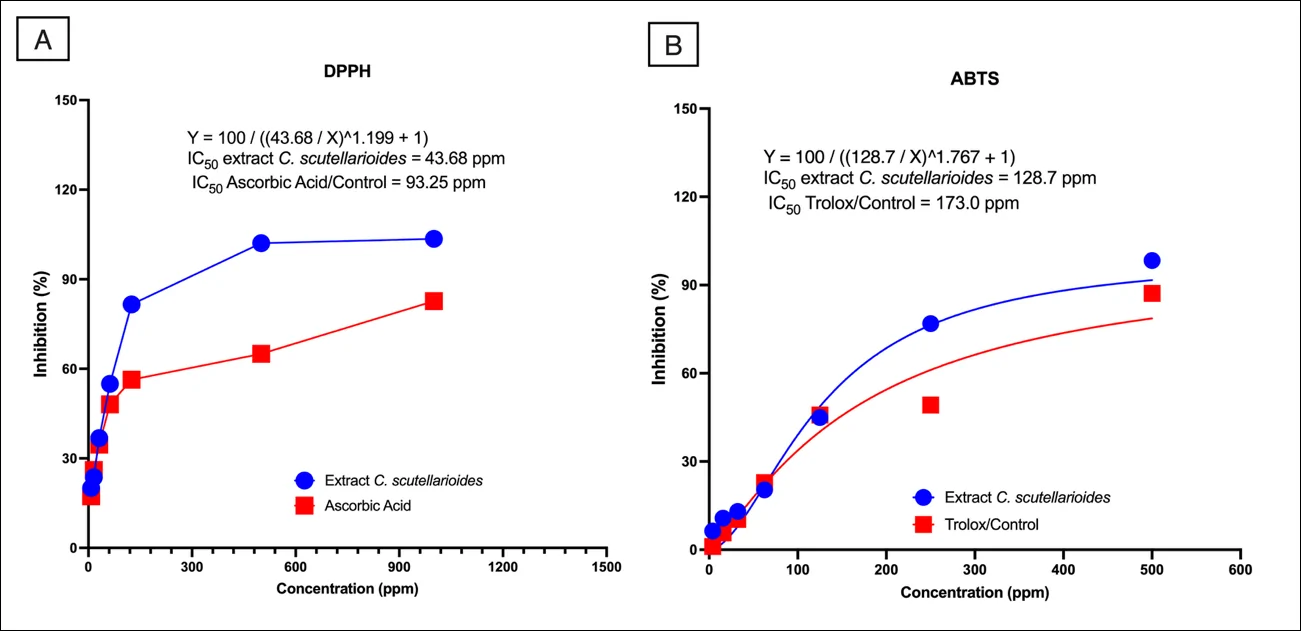
Antioxidant activity of the ethanol extract of Miana leaves. (A) 2,2-Diphenyl-1-picrylhydrazyl (DPPH) radical scavenging assay. (B) 2,2′-Azino-bis(3-ethylbenzothiazoline-6-sulfonic acid) (ABTS) radical scavenging assay. Data are presented as the mean of three independent replicates.

The ethanol extract of Miana leaves demonstrated free radical scavenging activity in both antioxidant assays (Figure 3). In the DPPH assay, the extract exhibited an IC₅₀ value of 43.68 ppm (95% CI: 30.98–60.90 ppm), indicating very strong antioxidant activity. The nonlinear regression model generated the equation:

Y = 100/[(43.68/X)^1.199^ + 1]

with a coefficient of determination (R²) of 0.9707. The antioxidant activity of the extract was greater than that of the positive control (ascorbic acid). Pearson's correlation analysis demonstrated a significant positive association between extract concentration and percentage radical inhibition (r = 0.8106, p = 0.03), indicating that radical scavenging activity increased with increasing extract concentration.

**In the ABTS assay, the extract exhibited an IC₅₀ of 128.7 ppm (95% CI:** 99.75–164.30 ppm), indicating moderate antioxidant activity. The nonlinear regression equation was:

Y = 100/[(128.7/X)^1.767^ + 1]

with an R² value of 0.9754. The antioxidant activity exceeded that of the positive control (Trolox). Pearson's correlation analysis revealed a strong positive relationship between extract concentration and radical inhibition (r = 0.9640, p < 0.001), confirming a consistent concentration-dependent response.

The dose–response curves for both assays exhibited the characteristic sigmoidal pattern of radical-scavenging activity, with inhibition increasing with increasing extract concentration. Comparison of the two assays demonstrated that the extract scavenged DPPH radicals more effectively than ABTS radicals, as indicated by an ABTS/DPPH IC₅₀ ratio of 2.95 (Figure 3).

## DISCUSSION

### Overview of the principal findings

This study comprehensively characterized the secondary metabolite profile of the ethanol extract of Miana leaves using LC–HRMS, evaluated its antibacterial activity against *Neisseria gonorrhoeae* and antifungal activity against *C. albicans* by determining the MIC and MFC values, and assessed its antioxidant activity using the DPPH and ABTS radical scavenging assays. Untargeted LC–HRMS analysis identified 243 annotated metabolites, including 20 major bioactive compounds belonging to the flavonoids (apigenin, kaempferol, apigetrin, cynaroside, diosmetin, and tangeretin), phenolic acids (caffeic acid), unsaturated fatty acids (stearidonic acid and α-eleostearic acid), terpenoids (lupeol, (+)-nootkatone, β-ionone, and (+)-ar-turmerone), diterpenoids (triptolide, kahweol, and carnosol), coumarins (coumarin), and alkaloids (betaine). Collectively, these findings demonstrate that the ethanol extract possesses a chemically diverse phytochemical composition that may contribute to its biological activities.

### Antioxidant activity of the extract

The ethanol extract of Miana leaves exhibited marked antioxidant activity in both radical scavenging assays. The DPPH assay yielded an IC₅₀ of 43.68 ppm, indicating very strong antioxidant activity, whereas the ABTS assay yielded an IC₅₀ of 128.7 ppm, corresponding to moderate antioxidant activity. The higher antioxidant activity observed in the DPPH assay, as reflected by an ABTS/DPPH IC₅₀ ratio of 2.95, suggests that the extract has greater affinity for lipophilic radicals than for hydrophilic radicals. This selectivity is likely attributable to the predominance of relatively lipophilic phytochemicals, particularly flavonoid aglycones such as apigenin and kaempferol and terpenoid compounds including lupeol and (+)-nootkatone, which exhibit strong hydrogen-donating and electron-transfer capacities toward DPPH radicals [40–42]. Furthermore, the significant positive Pearson correlation between extract concentration and radical-scavenging activity confirmed a concentration-dependent antioxidant response, indicating that the observed antioxidant activity was attributable to the extract's phytochemical constituents rather than analytical artifacts [43].

Beyond direct radical scavenging, the extract's antioxidant properties may enhance its antimicrobial effects through complementary biological mechanisms. Phenolic compounds, including caffeic acid and flavonoids, are known to modulate oxidative balance during microbial infection by scavenging excessive free radicals while simultaneously disrupting microbial redox homeostasis [44, 45]. Pakadang *et al.* [20] similarly reported that Miana leaf extract exhibits both antioxidant and antibacterial activities, supporting the present findings that these biological activities arise from the complex interactions among multiple phytochemical constituents rather than from a single active compound. Because oxidative stress contributes substantially to tissue injury, inflammation, and impairment of mucosal defense mechanisms during genital infections, the combination of antimicrobial and antioxidant activities may provide therapeutic advantages by simultaneously reducing microbial burden and limiting oxidative tissue damage [19, 27]. Likewise, Ślusarczyk *et al.* [15] reported that *Coleus* species exhibit high antioxidant activity associated with abundant flavonoids and phenolic compounds, which is consistent with the present LC–HRMS analysis, which demonstrates appreciable quantities of kaempferol, apigenin, caffeic acid, and related antioxidant metabolites.

### Antimicrobial activity of the extract

The ethanol extract demonstrated antifungal activity against *C. albicans*, with MIC and MFC values of 62,500 ppm (62.5 mg/mL), and antibacterial activity against *N. gonorrhoeae*, with MIC and MBC values of 100,000 ppm (100 mg/mL). The identical MFC/MIC and MBC/MIC ratios of 1.0 indicate that the extract exerted fungicidal and bactericidal activities at their respective MICs. Although these findings confirm the dual antimicrobial potential of the extract, the MIC values were considerably higher than those generally regarded as indicative of potent antimicrobial activity for crude plant extracts (MIC <0.1 mg/mL, highly active; 0.5–1.0 mg/mL, moderately active; 1.0–8.0 mg/mL, weakly active; >8–10 mg/mL, low biological relevance) [46, 47]. Therefore, despite demonstrating measurable antimicrobial activity, the crude ethanol extract exhibited relatively weak potency against the two target pathogens when compared with established antimicrobial agents.

The relatively high MIC values may be explained by several biological and physicochemical factors. First, crude plant extracts contain complex mixtures in which active constituents are substantially diluted by inactive metabolites, thereby reducing the apparent antimicrobial potency of the preparation. Second, the outer lipooligosaccharide membrane of *N. gonorrhoeae* represents an effective permeability barrier that limits the penetration of hydrophobic phytochemicals. Likewise, the cell wall architecture of *C. albicans* restricts the diffusion of many polar macromolecular constituents, thereby reducing intracellular accumulation of active compounds [48, 49]. Experimental artifacts were minimized by maintaining the final DMSO concentration below 1% (v/v), confirming complete solubility of the extract before inoculation, and demonstrating the absence of microbial inhibition in the vehicle controls [50, 51]. These methodological controls support the conclusion that the observed antimicrobial effects resulted from genuine biological activity rather than nonspecific physicochemical influences. Nevertheless, the relatively high effective concentrations indicate that bioassay-guided fractionation and purification of active constituents will be necessary before therapeutic application can be considered [52].

### Novelty and significance of the study

The present study provides several important advances over previous investigations of Miana. To our knowledge, this is the first study to combine comprehensive untargeted LC–HRMS metabolomic profiling with standardized CLSI-guided antimicrobial susceptibility testing against both *N. gonorrhoeae* and *C. albicans*. In addition to confirming dual antimicrobial and antioxidant activities, untargeted metabolomic analysis identified several bioactive metabolites, including triptolide, tangeretin, and betaine, that have not been previously reported in Miana. Furthermore, the use of an Orbitrap LC–HRMS platform with Metabolomics Standards Initiative-compliant compound annotation at a mass accuracy of <5 ppm provides substantially greater metabolomic coverage than earlier studies relying primarily on thin-layer chromatography or spectrophotometric phytochemical analyses [21]. From a microbiological perspective, the application of CLSI-guided broth microdilution with CO₂-supplemented incubation and confirmation on chocolate agar for *N. gonorrhoeae* represents a rigorous methodological approach that has rarely been applied in antimicrobial investigations of medicinal plant extracts. Consequently, this study establishes a robust phytochemical and microbiological reference for future investigations aimed at developing standardized phytopharmaceutical products targeting genital pathogens.

### Biological significance of the identified bioactive compounds

The predominance of flavonoids in the ethanol extract is consistent with the findings of Kueakulpattana *et al.* [10], who identified flavonoids as the principal bioactive constituents of *Coleus* spp. with antimicrobial activity mediated through inhibition of bacterial DNA gyrase and topoisomerase IV. Apigenin has been reported to exhibit broad-spectrum antibacterial activity by disrupting bacterial membrane integrity and inhibiting biofilm formation [53]. Similarly, kaempferol exhibits antifungal activity by inhibiting ergosterol biosynthesis in fungal cell membranes, a mechanism comparable to that of azole antifungal agents but with lower reported toxicity [19, 54, 55]. The detection of flavonoid glycosides, including apigetrin and cynaroside, suggests that these compounds function as storage and transport forms of flavonoids within plant tissues. Following hydrolysis by microbial enzymes or under acidic physiological conditions, these glycosides may release the corresponding aglycones, thereby enhancing the oral bioavailability and biological activity of the extract [40–42, 44]. Bismelah *et al.* [28] identified quercetin as the predominant constituent of *P. scutellarioides* and reported antibacterial activity against *Staphylococcus aureus* and *Pseudomonas aeruginosa* at substantially lower MIC values than those observed in the present study, likely attributable to differences in extraction methods, phytochemical composition, and the susceptibility of the target microorganisms.

Among the phenolic constituents, caffeic acid is recognized for its antimicrobial activity through induction of oxidative damage to microbial lipids, proteins, and nucleic acids by modulating intracellular ROS production [43, 45]. The unsaturated fatty acids stearidonic acid and α-eleostearic acid, detected at RT of 13.641 and 14.124 min, respectively, have been reported to disrupt phospholipid bilayers, thereby increasing membrane permeability and ultimately causing osmotic cell lysis [56–58]. Desbois and Smith [56] demonstrated that long-chain unsaturated fatty acids effectively inhibit *N. gonorrhoeae* through hydrophobic interactions with the outer membrane of Gram-negative bacteria, providing further support for the antibacterial activity observed in the present investigation.

Terpenoid constituents identified in the extract may also contribute substantially to its antimicrobial properties. Lupeol has been reported to disrupt fungal cell membrane fluidity and inhibit the dimorphic transition of *Candida* spp. from yeast to invasive hyphal forms [59–62]. Likewise, (+)-nootkatone and β-ionone are volatile terpenoids that rapidly penetrate microbial cells, disrupt mitochondrial function, deplete intracellular adenosine triphosphate, and ultimately induce cell death [63]. The identification of these compounds supports the hypothesis that multiple phytochemical classes collectively contribute to the observed antimicrobial activity through complementary mechanisms of action.

The diterpenoids identified in the present study further expand the biological relevance of the metabolomic profile. Triptolide has been reported to exhibit potent antifungal activity against fluconazole-resistant *Candida* spp. by inhibiting the calcineurin signaling pathway and inducing apoptosis in fungi [60, 64]. Carnosol exerts antimicrobial effects through a dual mechanism: inhibition of microbial efflux pumps and modulation of quorum-sensing pathways, thereby suppressing the expression of bacterial virulence factors [64–66]. Kahweol possesses a characteristic furan-diterpenoid structure that exhibits selective antibacterial activity against Gram-positive bacteria and moderate activity against Gram-negative organisms by inhibiting peptidoglycan biosynthesis [67, 68]. In addition, the repeated detection of coumarin peaks at multiple RT values suggests the presence of structurally related coumarin derivatives that interfere with microbial DNA replication through DNA intercalation and inhibition of topoisomerase enzymes [69]. Betaine, which exhibited one of the highest signal intensities, functions primarily as an osmoprotectant and methyl donor and may indirectly contribute to antimicrobial activity by modulating microbial oxidative stress responses [70].

### Differential antimicrobial susceptibility of the test microorganisms

The ethanol extract exhibited greater antifungal activity against *C. albicans* than antibacterial activity against *N. gonorrhoeae*, as reflected by MIC values of 62,500 and 100,000 ppm, respectively. This difference can be explained by fundamental structural differences between fungal and bacterial cells. The fungal cell membrane contains ergosterol, which has a relatively high affinity for hydrophobic flavonoids and terpenoids such as lupeol and apigenin, thereby facilitating intracellular accumulation of these bioactive compounds [71]. In contrast, *N. gonorrhoeae*, as a Gram-negative bacterium, possesses an outer lipooligosaccharide membrane that restricts penetration of hydrophobic phytochemicals, thereby reducing antimicrobial efficacy and increasing the concentration required for growth inhibition [72].

The identical MIC and MFC or MBC values obtained for both microorganisms (MFC/MIC or MBC/MIC ratio = 1.0) indicate that the extract exerted cidal rather than static antimicrobial effects at the MIC. Such cidal activity is advantageous because complete elimination of viable microorganisms may reduce the likelihood of persistent infection and the emergence of antimicrobial resistance [37, 39]. Bismelah *et al.* [28] reported that *P. scutellarioides* extract exhibited an MFC/MIC ratio of 2.0 against periodontal bacteria, which differs from the ratio observed in the present study. These differences are likely associated with variations in microbial susceptibility, the geographical origin of the plant material, the harvest season, extraction procedures, and the resulting phytochemical composition.

Previous investigations of Miana and *P. scutellarioides* have consistently reported lower MIC values against gram-positive and periodontal bacteria than against *N. gonorrhoeae* and *C. albicans*. This discrepancy may be attributed to three principal factors: the intrinsic permeability barrier of gram-negative bacteria [25, 28], differences in the susceptibility of the target microorganisms, and the use of crude extracts in the present study compared with partially purified fractions evaluated in previous investigations [28]. Importantly, the present study provides the first CLSI-standardized quantitative MIC and MFC values for these two clinically important genital pathogens together with comprehensive untargeted metabolomic profiling of Miana, thereby providing a valuable reference for future phytopharmaceutical research.

### Clinical relevance and future prospects

Compared with the positive controls, the crude ethanol extract exhibited substantially lower antimicrobial potency, being approximately 31,250-fold less active than nystatin against *C. albicans* and approximately 1,600,000-fold less active than vancomycin against *N. gonorrhoeae*. Nevertheless, these comparisons should be interpreted with caution because the extract is a complex mixture of phytochemicals rather than purified antimicrobial compounds (Table 3). Previous studies by Nguyen and Bhattacharya [74] and Vijayakumar *et al.* [75] demonstrated that flavonoids such as quercetin may act synergistically with conventional antibiotics by inhibiting microbial efflux pumps and modulating antimicrobial resistance mechanisms. Consequently, Miana leaf extract or its purified constituents may have greater potential as adjunctive therapies than as standalone antimicrobial agents.

**Table 3:** Comparison of the present study with previously published antimicrobial studies on Coleus scutellarioides and Plectranthus scutellarioides.

**Reference**	**Extract**	**Target microorganism**	**MIC (mg/mL)**	**Method**	**Major compounds identified**	**Principal findings**
Present study	96% ethanol (maceration)	*Neisseria gonorrhoeae* (ATCC 43069P), Candida albicans (ATCC 10231)	100; 62.5	CLSI broth microdilution	243 metabolites identified by LC–HRMS, including triptolide, carnosol, kahweol, kaempferol, and apigenin	Dual antimicrobial activity; DPPH IC₅₀ = 43.68 ppm; ABTS IC₅₀ = 128.7 ppm
[28]	Ethanol extract of P. scutellarioides leaves	Streptococcus oralis, Porphyromonas gingivalis, Aggregatibacter actinomycetemcomitans	1.56–3.13	Agar diffusion and MIC	Quercetin	Antibacterial activity reported
[17]	Ethyl acetate extract of Coleus spp.	Staphylococcus aureus	Not quantified	Disk diffusion	Flavonoids (qualitative)	Antibacterial activity reported
[73]	Ethanol extract of Miana leaves	C. albicans	In vivo only	Murine vulvovaginal model	Not characterized	Antifungal activity reported
[20]	Miana extract	Bacteria (unspecified)	Reported	Nonstandardized	Not characterized	Antibacterial activity reported
[15]	Coleus amboinicus extracts	Not evaluated	—	HPLC	Flavonoids and terpenoids	High antioxidant activity reported

MIC = Minimum inhibitory concentration, ATCC = American Type Culture Collection, CLSI = Clinical and Laboratory Standards Institute, DPPH = 2,2-diphenyl-1-picrylhydrazyl, IC₅₀ = half-maximal inhibitory concentration, ABTS = 2,2'-azino-bis(3-ethylbenzothiazoline-6-sulfonic acid), HPLC = High-performance liquid chromatography.

The dual activity of the extract against *N. gonorrhoeae* and *C. albicans* is of particular clinical interest because these pathogens frequently occur as coinfections that require simultaneous antibacterial and antifungal treatment [6, 73]. Although the observed cidal activity provides an encouraging pharmacological basis for further investigation, cytotoxicity toward mammalian cells and determination of the selectivity index (SI = CC₅₀/MIC) are essential before therapeutic applications can be proposed [76]. Bioassay-guided fractionation represents the most immediate strategy for concentrating active constituents and improving antimicrobial potency.

Future investigations should focus on isolation and structural characterization of the principal bioactive compounds, evaluation of synergistic interactions with ceftriaxone against *N. gonorrhoeae* and fluconazole against *C. albicans* using checkerboard and time-kill assays, and validation of antimicrobial efficacy using appropriate *in vivo* infection models [75]. Additional studies should include cytotoxicity assessment in vaginal epithelial or Vero cells, determination of the selectivity index, comparative LC–HRMS fingerprinting of Miana collected from different geographical regions, development of topical formulations such as creams, gels, and vaginal ovules, and ultimately phase I–II clinical trials to establish safety and therapeutic efficacy. Furthermore, proteomic and transcriptomic approaches may provide valuable insights into the molecular mechanisms underlying the antimicrobial activity of the identified phytochemicals.

### Study limitations

Several limitations should be considered when interpreting the findings of this study. First, the antimicrobial activity was evaluated using a crude ethanol extract, in which the concentrations of individual bioactive compounds were relatively low because of dilution within a complex phytochemical matrix. Consequently, the MIC and MFC/MBC values obtained may underestimate the antimicrobial potential of the purified active constituents. Second, although comprehensive untargeted LC–HRMS profiling successfully identified 243 metabolites, the study did not isolate or quantify the contribution of individual compounds to the observed antimicrobial and antioxidant activities. Therefore, the biological activities reported are likely attributable to synergistic interactions among multiple phytochemicals rather than to single constituents.

Third, the study was limited to *in vitro* antimicrobial and antioxidant assays. Although these findings provide valuable preliminary evidence, they do not fully represent the complex physiological environment encountered *in vivo*, where host immune responses, tissue penetration, metabolism, and pharmacokinetic behavior influence therapeutic efficacy. Furthermore, cytotoxicity toward mammalian cells was not evaluated. At the MIC values obtained (62.5 mg/mL for *C. albicans* and 100 mg/mL for *Neisseria gonorrhoeae*), potential nonselective effects on host cells cannot be excluded. Therefore, determination of the half-maximal cytotoxic concentration (CC₅₀) and the selectivity index (SI = CC₅₀/MIC) using appropriate mammalian cell lines, such as vaginal epithelial or Vero cells, is essential before therapeutic applications can be proposed [76].

Another limitation is that antimicrobial activity was evaluated only against standard reference strains. Clinical isolates, particularly multidrug-resistant strains, may exhibit different susceptibility profiles. In addition, although methodological measures were implemented to minimize physicochemical artifacts, the relatively high MIC values observed indicate that further purification of the extract is necessary to improve antimicrobial potency and facilitate therapeutic application.

### Future research perspectives

Future investigations should focus on bioassay-guided fractionation to isolate and characterize the individual phytochemicals responsible for the extract's antimicrobial activity. Purification of these compounds may substantially reduce the effective inhibitory concentrations while improving selectivity toward pathogenic microorganisms. Synergistic interactions between purified compounds and conventional antimicrobial agents, including ceftriaxone against *N. gonorrhoeae* and fluconazole against *C. albicans*, should be evaluated using checkerboard assays, fractional inhibitory concentration indices, and time-kill curve analyses to determine their potential as adjunctive therapies against antimicrobial-resistant pathogens [75].

Comprehensive preclinical investigations are also warranted, including cytotoxicity studies, pharmacokinetic analyses, pharmacodynamic evaluations, and efficacy testing in appropriate *in vivo* infection models. Comparative LC–HRMS metabolomic fingerprinting of Miana accessions collected from different geographical regions would improve phytochemical standardization and facilitate identification of chemical biomarkers for quality control of phytopharmaceutical products [25]. Development of advanced drug delivery systems, including polymeric nanoparticles, liposomes, and other nanoformulations, may improve mucosal penetration, prolong retention at the site of infection, and enhance antimicrobial efficacy while reducing the required therapeutic dose [77, 78]. Ultimately, well-designed clinical trials will be necessary to establish the safety, efficacy, and clinical applicability of standardized Miana-based formulations for the management of gonorrhea, vulvovaginal candidiasis, and mixed genital infections.

The identification of reproducible metabolomic fingerprints by LC–HRMS also provides an important platform for standardizing herbal preparations and establishing quality control parameters to ensure batch-to-batch consistency. Integration of metabolomic, transcriptomic, and proteomic approaches would further clarify the molecular mechanisms of action of the identified phytochemicals and facilitate the rational development of evidence-based phytopharmaceutical products.

## CONCLUSION

This study provides the first comprehensive integration of untargeted LC–HRMS metabolomic profiling with standardized CLSI-guided antimicrobial susceptibility testing and antioxidant evaluation of ethanol extracts of Miana leaves against the clinically important genital pathogens *Neisseria gonorrhoeae* and *C. albicans*. Untargeted LC–HRMS identified 243 annotated metabolites, including 20 major bioactive compounds from flavonoids, phenolic acids, terpenoids, diterpenoids, coumarins, unsaturated fatty acids, and alkaloids, thereby establishing a comprehensive phytochemical profile of the extract. The extract demonstrated fungicidal activity against *C. albicans* (MIC/MFC, 62,500 ppm [62.5 mg/mL]) and bactericidal activity against *N. gonorrhoeae* (MIC/MBC, 100,000 ppm [100 mg/mL]), with MFC/MIC and MBC/MIC ratios of 1.0. In addition, the extract exhibited very strong antioxidant activity in the DPPH assay (IC₅₀, 43.68 ppm) and moderate antioxidant activity in the ABTS assay (IC₅₀, 128.7 ppm), indicating its dual antimicrobial and antioxidant potential.

The principal strength of this study lies in the combination of high-resolution untargeted metabolomic characterization and standardized antimicrobial susceptibility testing, providing quantitative microbiological data alongside detailed phytochemical information. The identification of several bioactive metabolites not previously reported in Miana further expands the current understanding of the phytochemical diversity of this medicinal plant and provides valuable reference data for future phytopharmaceutical standardization.

Despite these promising findings, the antimicrobial potency of the crude ethanol extract remained relatively low compared with conventional antimicrobial agents, indicating that therapeutic application of the unfractionated extract is currently limited. Furthermore, the absence of cytotoxicity evaluation, determination of the selectivity index, and *in vivo* efficacy studies precludes definitive conclusions regarding its clinical applicability.

Future investigations should prioritize bioassay-guided fractionation to isolate the principal active constituents, evaluation of synergistic interactions with conventional antimicrobial agents, comprehensive cytotoxicity and pharmacokinetic studies, and validation using appropriate *in vivo* infection models. Standardization of extracts through LC–HRMS chemical fingerprinting and development of optimized pharmaceutical formulations will also be essential steps toward translation into clinical applications.

Overall, the findings demonstrate that ethanol extracts of Miana leaves possess measurable dual antimicrobial and antioxidant activities supported by a diverse metabolomic profile. Although further optimization is required to improve antimicrobial potency and establish safety, this study provides a robust scientific foundation for the future development of standardized Miana-based phytopharmaceutical products as complementary therapeutic candidates for the management of gonorrhea, candidiasis, and mixed genital infections.

## DATA AVAILABILITY

The data generated during the study are included in the manuscript.

## AUTHORS’ CONTRIBUTIONS

MBK: Conceptualization, methodology, validation, formal analysis, investigation, data curation, original draft preparation, supervision, project administration, and funding acquisition. FMS: Conceptualization, methodology, and supervision. TRA: Methodology, validation, resources, and review and editing of the manuscript. DM: Software, formal analysis, resources, and data curation. DD: Investigation, data curation, and visualization. YM: Software, formal analysis, investigation, and visualization. All authors have read and approved the final manuscript.
